# Impact of temporomandibular disorders on the stomatognathic system in children

**DOI:** 10.4317/medoral.22000

**Published:** 2017-10-21

**Authors:** Kranya-Victoria Díaz-Serrano, Taiana de Melo Dias, Paulo Vasconcelos, Luiz-Gustavo Sousa, Selma Siéssere, Simone Regalo, Marcelo Palinkas

**Affiliations:** 1 Department of Pediatric Clinics, School of Dentistry of Ribeirão Preto, University of São Paulo-USP, Ribeirão Preto, SP, Brazil; 2 Department of Morphology, Physiology and Basic Pathology, School of Dentistry of Ribeirão Preto, University of São Paulo-USP, Ribeirão Preto, SP, Brazil; 3 ….

## Abstract

**Background:**

To evaluate the EMG activity and thickness of right masseter (RM), left masseter (LM), right temporal (RT) and left temporal (LT) muscles and bite force in children with temporomandibular disorders (TMD).

**Material and Methods:**

Forty five children (mean age 8.8 years; 22 boys and 23 girls) were examined on the basis of the RDC/TMD and the Faces Pain Scale-Revised (FPS-R) was used to determine the level of severity of the signs and symptoms of TMD, resulting in four groups: GI - without TMD (n=10); GII - with mild TMD (n=18), GIII: with moderate TMD (n=12) and GIV: with severe TMD (n=5). The data of electromyographic activity, maximum bite force and muscle thickness were tabulated and submitted to statistical analysis (ANOVA, *P*≤0.05).

**Results:**

Children with TMD signs and symptoms had lower EMG activity than children of the control group. There was significant difference among the groups for the LT at rest (*P*=0.01), right (*P*=0.03) and left (*P*=0.05) laterality, and for the LM (*P*=0.01) and LT (*P*=0.03) muscles in maximum voluntary contraction. There were no statistically significant differences among the groups regarding muscle thickness. The bite force was lower in the TMD groups than children of the control group, with significant statistical difference for the right region (*P*=0.03).

**Conclusions:**

The severity of TMD signs and symptoms affected the EMG activity and the molar bite force in children. However, structural changes in the thickness of masticatory muscles are not perceptible in children with TMD signs and symptoms.

** Key words:**Children, temporomandibular disorders electromyographic, bite force, ultrasound.

## Introduction

Temporomandibular disorders is a collective term used to describe a cluster of disorders characterized by clinical signs and symptoms involving the temporomandibular joint, masticatory muscles and/or associated structures (1-3).

Local factors like occlusal interferences, psychological factors influencing the psychomotor activities, traumas and systemic factors have an important role on temporomandibular disorders multifactorial etiology (4).

TMD has primarily been referred to as a condition affecting adults, but epidemiological studies have reported signs and symptoms of this stomatognathic disorder in children and adolescent (5) including temporomandibular joint noises during mandibular function, deviation of the mandible on opening/closing, mouth opening restriction, headaches, earaches, pain on the temporomandibular joint, masticatory and facial muscles (6).

Masticatory muscles have synergic functional activities that are responsible for temporomandibular joint movement or even stabilization of that (7). Thus, electromyographic and ultrasound imaging analyses of masticatory muscles offer important information to evaluate the functional performance and pathological conditions of the stomatognathic system, as well as the maximal bite force (8,9).

Considering that morpho-functional alterations can be observed in individuals with temporomandibular disorders, this study evaluated the EMG activity and the thickness of masseter and temporalis muscles and the maximal bite force in children with different severity levels of temporomandibular disorders compared to children without disorders.

## Material and Methods

- Sample

Ninety-three children, aged 7 to 11 years (mean age 8.8 years), under routine dental care at the Pediatric Dentistry Clinic, University of São Paulo, without prefe-rence for gender or ethnic group, were considered as eligible for the study.

Children were not included in the study if they presented systemic diseases, ongoing treatment with medications that may affect muscular activity such as antihistaminic, anxiolytic, homeopathic or other drugs with suppressive action on the central nervous system had an uncooperative behavior or presented history of trauma, dental pain, orthodontic treatment, otorhinolaryngological treatment or speech therapy.

Forty-five children participated in the study (22 boys and 23 girls). After approval of the research project by the Institutional Ethics Committee of the School of Dentistry of Ribeirão Preto, University of São Paulo, Brazil (process n. 2007.1.1366.58.9), the parents/caregivers were fully informed about the procedures, possible discomforts and risks, as well as the potential benefits, and signed an informed consent form authorizing the children’s participation. The research was conducted in accordance with the Declaration of Helsinki.

The participants were further subjected to clinical examination, for determine the presence and intensity of temporomandibular disorders signs and symptoms and electromyographic activity, thickness masticatory muscles and maximal bite force, were evaluated.

- Clinical Examination 

A single trained examiner evaluated all children. On the basis of Axis I of the Research Diagnostic Criteria for TMD (RDC/TMD) (10), the following signs and symptoms were recorded: pathways of habitual mouth opening and closing: mandibular deviation to the left or right (by measuring the lower midline distance between the lower and upper central incisors, in relation to the upper midline), pain and joint noises associated with the temporomandibular joint, tenderness and pain on the temporal and masseter muscles were evaluated by bilateral palpation (11). The child was asked about the difference in sensitivity between the right and left sides. The Faces Pain Scale-Revised (FPS-R), which shows a close linear relationship with visual analogue pain scales across the age range of 4-16 years, was used in this study to determine the level of severity of pain. It is a self-report measure of pain intensity combining drawings of face expressions and numerical self-rating scales (0 - 10). It shows a series of six faces that illustrate increasing levels of pain, from the first face with no pain “0” and the last face showing the most severe pain “10”.

Thus, children were distributed in four groups based on the level of severity of signs and symptoms of temporomandibular disorders: GI: without TMD (n=10), GII: mild TMD (n=18), GIII: moderate TMD (n=12) and GIV: Severe TMD (n=5) with severe TMD.

The EMG signals, ultrasound images and bite force measures were obtained in a calm and quiet environment with the children sitting upright on a comfortable office-like chair, with the sole of the feet contacting the ground, the arms extended along the body and the hands lying on their thighs. The head was maintained upright with the occlusal plane parallel to the ground.

- EMG analysis

The EMG analysis was performed using a electromyographer Myosystem-Br1 (DataHomins Ltda. Uberlândia, MG, Brazil) with simultaneous acquisition, common grounding to all channels, low-pass filters of 10 Hz to 5 KHz; channel input impedance of 10 G ohms in differential mode, 12 bites of dynamic resolution range, amplitude band of -10 V to +10 V, and channel sampling frequency of 2 KHz. Using the software Myosystem I (v. 3.56), the signals were visualized, processed, digitized and then analogically amplified with a ×1,000 gain, filtered by a 0.01-1.5 kHz bandpass filter and sampled by a 12-b A/D converter with an acquisition frequency of 2 kHz.

Surface differential active electrodes were used in the study. The skin region where electrodes were placed was previously cleaned with isopropyl alcohol and shaved when necessary. The differential active electrodes were positioned in the ventral region of masseter and temporalis muscles.

The correct position of electrodes was determined by digital palpation. The electrodes were fixed with adhesive bandage tape, with the longest extension of the bars perpendicular to the direction of the muscle fibers. A 3-cm-diameter stainless steel circular electrode was fixed on the skin of the frontal bone region and served as a reference electrode.

The EMG activity of the masseter and temporalis muscles was recorded at rest (5s) and during activities involving the active participation of these muscles right (5s) and left (5s) laterality, protrusion (5s); dental clenching in maximum voluntary contraction (4s) and maximal voluntary contraction with Parafilm M® (Pechinery Plastic Packaging, Batavia, IL, USA) (4s).

- Muscle Thickness analysis

Masseter and temporalis muscle thickness was measured during relaxation and maximum voluntary contraction using a portable high-resolution modular ultrasound device (SonoSite, Inc. Worldwide Headquarters, Bothell, WA, USA) with a high-resolution real-time 56 mm/10-MHz linear-array transducer, placed transversally to the muscle fibers. Bilateral ultrasound images of the masseter and temporalis muscles were obtained at rest and during dental clenching in maximal voluntary contraction. Three examinations were done for each of these conditions with 2-min intervals between the measurements.

- Bite force analysis

Bite force records were obtained by a digital dynamometer, model IDDK (Kratos, Cotia, São Paulo, Brazil), with a 980.665 N capacity and adapted to the mouth. The apparatus has a “set-zero” key, which allows exact control of the values obtained and also “peak” registers, which facilitate recording of the maximal force during measures.

It has two arms with plastic disks on each end, over which the force to be measured is applied. Its high-precision charge cell and electronic circuit to indicate force supply precise measures easily viewed on a digital display. The dynamometer was cleaned with alcohol, and disposable latex finger cots (Wariper-SP) were positioned on the biting arms as a biosecurity measure.

The participants were given detailed instructions and bite tests were performed before the actual recordings were made in order to ensure the reliability of the procedure. The volunteers were then asked to bite the dynamometer three times with maximal force, with a two-minute rest interval between records. Evaluations were performed in the first molar, left and right regions.

- Statistical Analysis 

Maximal voluntary contraction with Parafilm M® was used for as the normalization factor of the EMG data. The EMG data , ultrasound images data and the values obtained for the maximum molar bite force were tabulated and analyzed statistically by ANOVA (*P*≤0.05) using SPSS software version 21.0 for Windows (SPSS Inc., Chicago, IL, USA).

## Results

Based on results from the RDC/TMD, children in GII, GIII and GIV predominately showed muscle disorders. Pain was reported by the subject in response to palpation of three or more of the following muscle sites (right side and left side count as separate sites for each muscle): posterior temporalis, middle temporalis, anterior temporalis, origin of masseter, body of masseter and insertion of masseter.

Children of GI, GII and GIII, were pain-free when unassisted mandibular opening of <40 mm was performed, as well as during the maximum assisted opening (passive stretch).

Crepitus was absent in all children. Reciprocal clicking in TMJ on both vertical range of motion, either opening or closing, which was reproduced on two of three consecutive trials, was observed in 60% of the total sample, and deflection during mandible opening of >2mm was also common in all groups (55,56%).

Severe muscle pain was the most common symptom in the children of GIV, with severe TMD. This fact certainly influenced the results, mainly referring to EMG activity and bite force. All of the signs and symptoms of TMD evaluated were more frequent in this group. Besides muscle ache, restriction of mandibular movement, as well as, arthralgia pain on the lateral pole in one or both joint sites during palpation of TMJ, was observed in 80% of the subjects of GIV.

 Table 1 showed that there was significantly greater (*P*≤0.05) activation of the right and left masseter muscles in the four groups evaluated. There was statistically significant difference among the groups for the LT at rest (*P*=0.01) and during right (*P*=0.03) and left (*P*=0.05) laterality, and for the LM (*P*=0.01) and LT (*P*=0.03) muscles in maximum voluntary contraction. All children presented EMG activity of the masseter and temporalis muscles in the mandibular rest position, and the children of the control group presented the highest values.

**Table 1:**
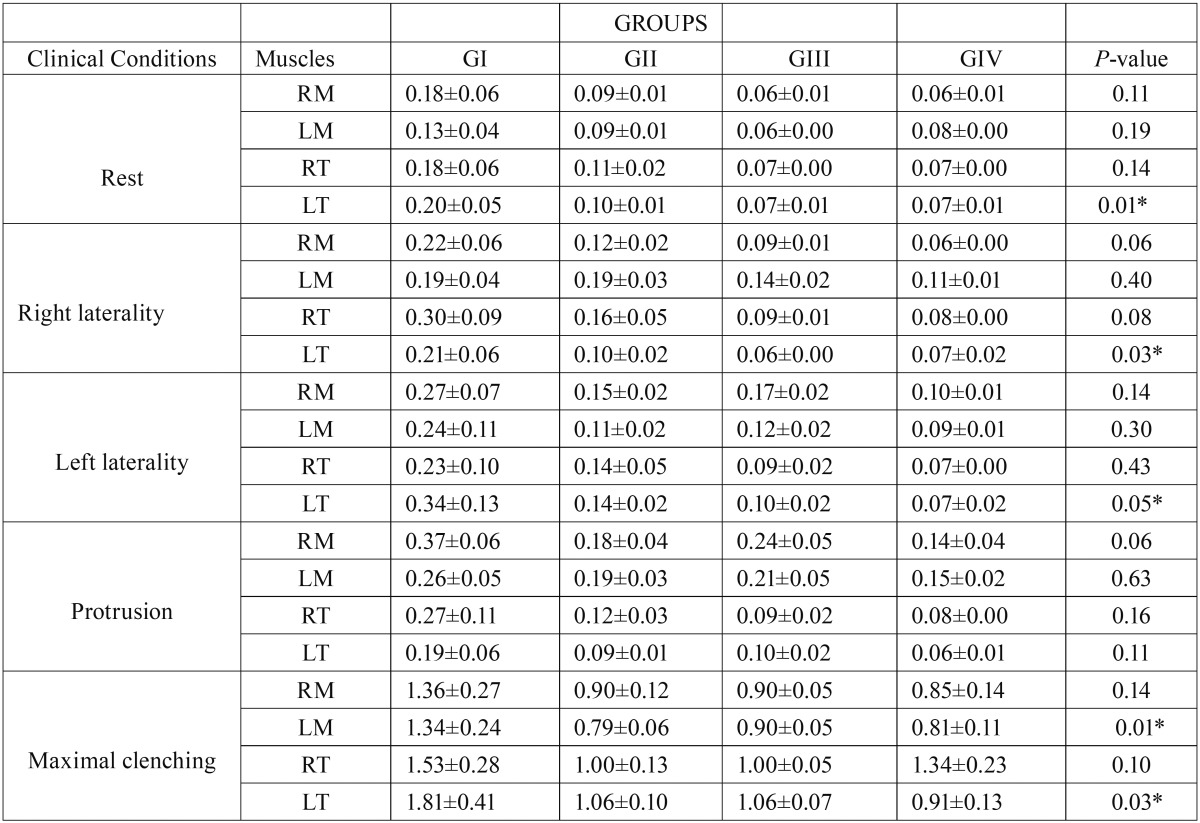
Means, standard errors (±) and statistical significance (*P*≤0.05*) of the normalized electromyographic data (µV) averages of the right masseter (RM), left masseter (LM), right temporalis (RT) and left temporalis (LT) for GI (without TMD), GII (with mild TMD), GIII (with moderate TMD) and GIV (with severe TMD) in the clinical conditions.

The averages of thickness of the masseter and temporalis muscles in the maximal voluntary contraction and rest for the groups are shown in  Table 2. There were no statistically significant differences among the groups (*P*≥0.05).

**Table 2:**
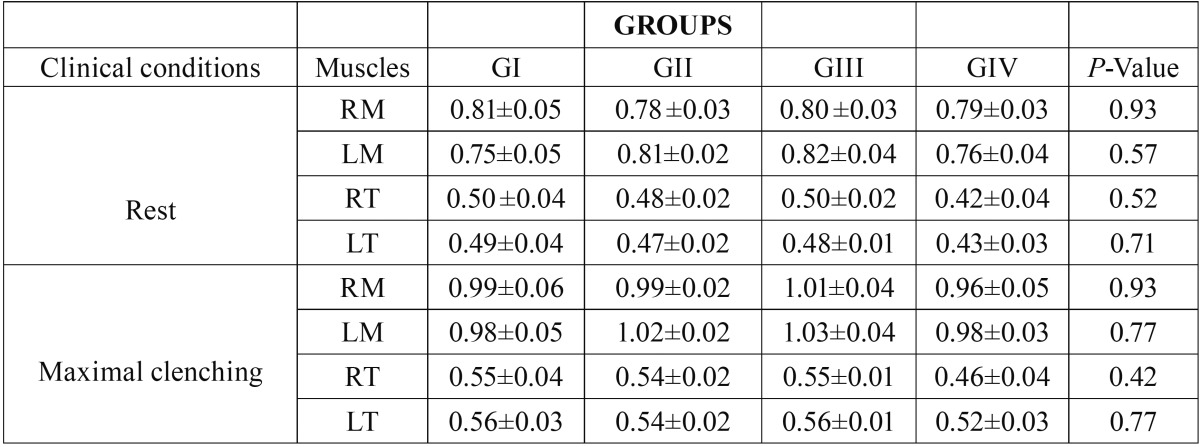
Averages means, standard errors (±) and statistical significance (*P*≤0.05*) of the thickness of the right masseter (RM), left masseter (LM), right temporalis (RT) and left temporalis (LT) during rest and dental clenching for GI (without TMD), GII (with mild TMD), GIII (with moderate TMD) and GIV (with severe TMD).

The average values of the maximum molar bite force are shown in  Table 3. There was a statistically significant difference between the analyzed groups on the left side (*P*≤0.05).

**Table 3:**
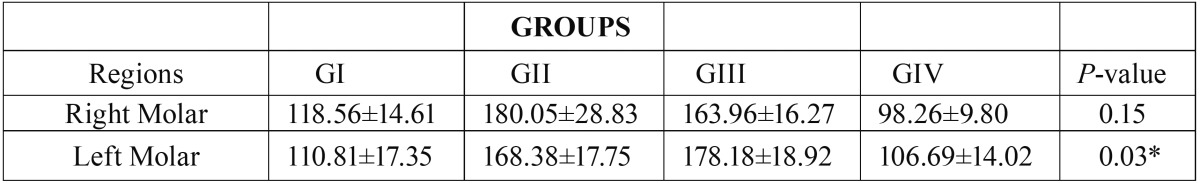
Means, standard errors (±) and statistical significance (*P*≤0.05*) of the right and left molar bite force (N) for for GI (without TMD), GII (with mild TMD), GIII (with moderate TMD) and GIV (with severe TMD).

## Discussion

In the present study, EMG analysis was used to evaluate the activity of masticatory muscles under different clinical situations in children. EMG activity during maximal voluntary contraction was also evaluated to normalize the data collected from the study sample (12,13), which is an attempt to minimize the alterations among the different records performed in the same individual or in different individual in order to make the obtained data reproducible (14).

The high-resolution ultrasound scans of the masseter and temporalis muscles provided an accurate and fast measurement of the thickness of these muscles, without exposing the children to radiation, as computed tomography, which makes ultrasound images analysis adequate for examination of masticatory muscles (15,16).

In the present study, all children presented EMG activity of the masseter and temporalis muscles in the mandibular rest position, and the children of the control group presented the highest values. In this clinical condition, no or minimal EMG activity should be recorded because the masticatory muscles must be relaxed with no contraction of motor units (17). This fact demonstrates that some mechanisms about the tonic activity of masticatory muscles in early periods of life are not yet fully understood and should be further elucidated.

According to the concepts of muscular neuroanatomic activation, greater EMG activity of the temporalis muscle occurs in the side that the mandible is moving (working side) during lateral mandibular excursions, while the masseter muscle in the contra-lateral side (non-working side) is more activated during this movement (18). In the present study, this pattern was observed only in the children of the control group, which demonstrates that even mild TMD signs and symptoms promote alterations in the patterns of muscular activation.

It is suggested that during mandibular protrusion, the masseter muscles should present greater EMG activity than the temporalis muscles, which was observed in the present study in all groups, deducing that the influence of TMD there is practically null (19).

The highest EMG activity of masseter and temporalis muscles is observed during maximal voluntary contraction in individuals with complete dentition and no mandibular disorders (20), which is in accordance with the findings of the present study. During maximal voluntary contraction, the maximum number of dental contacts is obtained, which represents an increase in the contact area in occlusion (21). In the present study the temporalis muscles presented higher EMG activity than the masseter muscles during maximal voluntary contraction. A possible explanation was the fact that our sample was predominantly composed of children in the mixed dentition, that is, interferences of oclusal contacts were present, even if they were physiological (22). Another hypothesis would be that children with temporomandibular disorders used the temporalis muscles during maximal voluntary contraction as result of nociceptive inputs to reduce painful symptomatology (23).

All ultrasound images showed that contracted muscles were thicker than relaxed muscles, which was an expected result and had been previously demonstrated (15). However, masseter muscles presented greater thickness than the temporalis muscles in all groups both at rest and during maximal voluntary contraction, which is in agreement with the findings of previous studies evaluating children in the mixed dentition phase (24).

We speculate that the structural changes on the thickness of the muscles, resultant of the presence of the TMD signs and symptoms and occlusal alterations, are not perceptible in children (25). Considering the age of the children in this study, it may be assumed that there had not been enough time in their life for the deleterious effects of temporomandibular disorders to cause measurable changes in muscle thickness.

The literature about the magnitude of bite force in the different dentition stages (early primary dentition, late primary dentition, early mixed dentition, late mixed dentition, and permanent dentition stages) isn’t enough (26). However, the age has been considered one of the factors that influence the magnitude of the bite force in individuals, as well as, the gender, height, craniofacial morphology, periodontal support of teeth, signs and symptoms of temporomandibular disorders, dental status (27), thickness and activity of the masticatory muscles, the condition of the child’s dentition and physiological development (15). Some studies show that bite strength is higher in males and increases with age and development of dentition (28). It is believed that this force is lower during the mixed dentition stage when compared to the permanent dentition stage (25).

It should be noted that the magnitude of the bite force varies when the characteristics of the craniofacial complex are abnormal, as in the case of occlusal changes (cases of cross bite and temporomandibular disorders) (29) and increasing stages of dental eruption in children (30) The increase in the number of occlusal contact during transition through the different dentition stages is correlated with bite force (31).

In this study, the results showed that individuals with mild and moderate signs and symptoms of TMD had higher values of bite force. This can be explained based on the assertion that individuals with TMD use a masticatory force comparatively higher than normal subjects during mastication (32).

In an evaluation of patients between 6 and 18 years of age, Pereira *et al.* (25) noticed no significant correlation between TMD and bite strength in the mixed dentition stage. However, the results obtained by this group when assessing individuals, particularly females, at the stage of permanent dentition showed a correlation between signs and symptoms of TMD. It is of note that could not been observed in the present investigation any difference in bite force between genders. Reports of muscle pain with a reduction of bite force in this study suggested that the painful symptoms impede individuals from exercising the maximal bite force (33).

Articular disorders may influence the muscular force realized in individuals with permanent dentition who had joint sounds and pain showed the lowest values of bite force (34). This is similar to the results of this study, in which lower values were obtained for the magnitude of bite force in the group that showed severe signs and symptoms of TMD. Therefore, it is reasonable to assume that pain in the masticatory muscles prevented the patients from exerting maximum bite force (35). Muscular tenderness had been considered one of the most prevalent clinical sign of the TMD in children (36).

According to Kogawa *et al.* (37), there is not sufficient knowledge about the mechanisms responsible for the differences in maximal bite force for individuals with muscle pain and changes in the temporomandibular joint. However, the results of this current study support the hypothesis that the strength of the bite can be affected by the presence of signs and symptoms of TMD.

## Conclusions

Based on the results of this study, the authors concluded that children of signs and symptoms of TMD showed morphological and functional alterations in the stomatognathic system, especially in electromyographic activity and maximum molar bite force.
